# Ceramide synthesis regulates biogenesis and packaging of exosomal MALAT1 from adipose derived stem cells, increases dermal fibroblast migration and mitochondrial function

**DOI:** 10.1186/s12964-022-00900-9

**Published:** 2023-08-24

**Authors:** Xaioyuan Kong, Niketa A. Patel, Charles E. Chalfant, Denise R. Cooper

**Affiliations:** 1 Department of Veterans Affairs, J.A. Haley Veterans Hospital, Research Service 151, Tampa, Fl 33711 USA; 2 Department of Molecular Medicine, Morsani College of Medicine, Tampa, USA; 3 Department of Cellular Biology, Microbiology and Molecular Biology, University of South Florida, Tampa, FL 33612 USA

**Keywords:** Adipose-derived stem cells, Cell migration scratch assay, Ceramide, Exosomes, Extracellular vesicles, Human dermal fibroblasts, lncRNA, MALAT1, Mitochondrial stress test, Sphingomyelinase

## Abstract

**Background:**

The function of exosomes, small extracellular vesicles (sEV) secreted from human adipose-derived stem cells (ADSC), is becoming increasingly recognized as a means of transferring the regenerative power of stem cells to injured cells in wound healing. Exosomes are rich in ceramides and long noncoding RNA (lncRNA) like metastasis-associated lung adenocarcinoma transcript 1 (MALAT1). We identified putative ceramide responsive cis-elements (CRCE) in MALAT1. We hypothesized that CRCE respond to cellular ceramide levels to regulate sEV MALAT1 packaging. MALAT1 levels by many cells exceed those of protein coding genes and it’s expression is equally high in exosomes. Ceramide also regulates exosome synthesis, however, the contents of exosome cargo via sphingomyelinase and ceramide synthase pathways has not been demonstrated.

**Methods:**

ADSC were treated with an inhibitor of sphingomyelinase, GW4869, and stimulators of ceramide synthesis, C2- and C6-short chain ceramides, prior to collection of conditioned media (CM). sEV were isolated from CM, and then used to treat human dermal fibroblast (HDF) cultures in cell migration scratch assays, and mitochondrial stress tests to evaluate oxygen consumption rates (OCR).

**Results:**

Inhibition of sphingomyelinase by treatment of ADSC with GW4869 lowered levels of MALAT1 in small EVs. Stimulation of ceramide synthesis using C2- and C6- ceramides increased cellular, EVs levels of MALAT1. The functional role of sEV MALAT1 was evaluated in HDF by applying EVs to HDF. Control sEV increased migration of HDF, and significantly increased ATP production, basal and maximal respiration OCR. sEV from GW4869-treated ADSC inhibited cell migration and maximal respiration. However, sEV from C2- and C6-treated cells, respectively, increased both functions but not significantly above control EV except for maximal respiration. sEV were exosomes except when ADSC were treated with GW4869 and C6-ceramide, then they were larger and considered microvesicles.

**Conclusions:**

Ceramide synthesis regulates MALAT1 EV content. Sphingomyelinase inhibition blocked MALAT1 from being secreted from ADSC EVs. Our report is consistent with those of MALAT1 increasing cell migration and mitochondrial MALAT1 altering maximal respiration in cells. Since MALAT1 is important for exosome function, it stands that increased exosomal MALAT1 should be beneficial for wound healing as shown with these assays.

Video Abstract

**Supplementary Information:**

The online version contains supplementary material available at 10.1186/s12964-022-00900-9.

## Background

Exosomes are ceramide-enriched small extracellular vesicles generated by inward budding of the endosomal membrane and secreted when the multivesicular endosomes fuse with the plasma membrane [1, 2]. These small vesicles, 40–160 nm, contain a cargo of protein factors, mRNA, long non-coding RNA (lncRNA), snRNA, tRNA and microRNA (miRNA) in addition to lipids, that are taken up by other cells to mediate intercellular communication [3]. Although miRNAs in exosomes have been widely investigated, lncRNA packaging and function in these vesicles is largely unknown. The lncRNA, metatasis-associated lung adenocarcinoma transcript 1 or MALAT1 is abundantly expressed at levels that are even higher than many protein coding genes, including β-actin or GAPDH by cells [4]. MALAT1 is processed into a 6.7 or 8.4 kb transcript from mice and humans [5]. We have shown that MALAT1 is one of the most abundant lncRNA identified in exosomes from human adipose derived stem cells (ADSC)[6]. Exosomes, when applied to various models of wound healing, increase the rate of cell migration in scratch assays and an in vivo model of ischemic wounds[7]. MALAT1 has numerous cellular functions including alternative splicing of pre-mRNA, control of gene expression, recruitment of transcription factors, and recruitment of competitive endogenous RNAs and miRNA [4]. MALAT1 can bind serine/arginine-rich (SR) splicing factors (SF) such as SRSF1 to promote cell proliferation[8]. In the case of cultured ADSC, most of the MALAT1 detected is exosomal suggesting that in stem cells it functions differently from its nuclear splicing action in cells[9]. When hADSC begin to differentiate to adipocytes, high levels of exosomal MALAT1 are no longer the case, and most MALAT1 is retained by pre-adipocytes and adipocytes. We wanted to determine how MALAT1 was destined for packaging into ADSC exosomes.

Exosomes are rich in ceramide [10], and MALAT1 contains multiple ceramide response cis-elements (CRCE) core motifs within it [11]. CRCE in RNA can regulate transcription, alternative splicing of transcripts, transport of proteins into vesicles, activity of enzymes, and secretion of exosomes [11–14]. We hypothesized that ceramide production played a role in the packaging and secretion of MALAT1. Exosome biogenesis is regulated in a ceramide-dependent manner dependent upon neutral sphingomyelinase (n-SMase)[10]. GW4869, an inhibitor of n-SMase1 and 2 reduces the release of proteolipid protein (PLP)-positive exosomes[2]. In the presence of GW4869, exosomes are not secreted but microvesicles, > 300 nm, are detected instead. Here, we report the effects of GW4869 treatment on exosomes and microvesicle function in cells. C2- and C6-ceramide incorporate into longer species of ceramide via de novo ceramide synthesis. This effect was investigated on MALAT1 levels in cells and exosomes and microvesicles, and on cell migration and mitochondrial function of human dermal fibroblasts (HDF) as a model for wound healing. Our results indicate that the loss and gain of MALAT1 levels in exosomes and microvesicles via ceramide synthesis has functional consequences for cell migration and mitochondrial function related to wound healing.

## Materials and methods

### Culture of adipose derived stem cells

Human adipose derived stem cells (hADSC) were purchased (Zen-Bio Inc., Durham, N.C., # ASC-F). The cells screened negative for HIV-1, HIV-2, HTLC-1, HTLV-2, Hep-B, Hep-C, and mycoplasma. HADSC were isolated from subcutaneous adipose tissue of normal, nondiabetic donors between 25 and 45 years of age undergoing elective surgery and with BMI < 23.3. Cells were cultured according to the manufacturer’s instructions.

### Collection of conditioned media (CM)

Exosomes and microvesicles were isolated from conditioned media (CM). For CM collection, hADSC were grown to 90% confluence in T75 flasks, media was replaced with serum-free mesenchymal stem cell basal media, with StemFlex medium kit (GibcoLifeTechnologies, Waltham, MA, # A33494-01) and CM was collected after 48 h.

For GW4869**-**CM, the hADSC cells were treated with GW 4869 (1 µM) (from Cayman Chemical, Ann Arbor, MI, # 13,127) in serum-free media for 48 h. For C2-ceramide CM, hADSC were treated with C2-ceramide (Cayman Chemical, # 62,510), a biologically active, cell permeable and less hydrophobic analog of natural ceramide, at 20 µM in serum-free media, 48 h. For C6-ceramide CM, hADSC were treated with C6-ceramide (Cayman Chemical, # 62,525) at 20 µM with serum-free media, 48 h.

### Isolation of exosomes and microvesicles

Exosomes and microvesicles were isolated using Exo-Spin Exosome Purification Kit (Cell Guidance System, St. Louis, MO, # EX01-25). Briefly, 45 ml of CM was transferred to a conical tube and centrifuged at 300 × g for 10 min to remove any unattached cells. The supernatant was transferred to a new tube and spun at 16,000 × g for 30 min to remove any remaining cell debris. Next, the supernatant was transferred to a new tube, and ½ volume of Exo-Spin Buffer was mixed with it and incubated at 4 °C overnight. This was then centrifuged at 16,000 × g for 1 h, carefully aspirated and the supernatant was discarded. The exosome-containing pellet was resuspended in 100 µl of PBS. The Exo-Spin Column was prepared following manufacturer’s instructions. The exosome containing pellet (100 µl) was carefully applied to the top of column and the column was placed into the waste tube. This was centrifuged at 50 × g for one min, and the eluate was discarded. The column was placed into a 1.5 ml microcentrifuge collection tube, and 200 µl of PBS was applied to the column and centrifuged at 50 × g for one min. The 200 µl eluate contained the purified sEV containing exosomes and microvesicles.

### Measurement of MALAT1 in cells and small extracellular vesicles (sEV)

Human ADSC were cultured in T-75 flasks. For CM collection, hADSC were grown to about 90% confluence, medium was replaced with serum-free mesenchymal stem cell basal medium, we used StemFlex medium kit (Gibco, # A33494-01) and CM (15 ml) were collected after 48 h. Total cellular RNA was isolated from sEV using RNeasy Mini Kit (Qiagen, Germantown, MD). One microgram of total RNA was used to synthesize complementary cDNA with iScript cDNA Synthesis Kit (BioRad, Hercules, CA, # 1,708,891 according to the manufacturer’s protocol. An iScript reaction was added to the reverse transcriptase with the sample in nuclease free water; a thermal cycler program was applied. Next, the Real-Time PCR reaction was performed using 2 µl of cDNA with 1 µl of 20X Taqman Human MALAT1 primer/probe set (Applied Biosystems, Foster City, CA, # Hs00273907_S1), 10 µL of TaqMan® Fast Advanced Master Mix (Applied Biosystems, Foster City, CA) and 7 µL of Nuclease-Free Water for a total volume of 20 uL. As an internal control, a second Real-Time PCR reaction was performed using 2 uL of cDNA with 1 µL 20X Taqman Human GAPDH primer/probe set (# Hs03929097_g1) Thermocycling conditions were as follows: 50 °C, 2 min; 95 °C, 10 min; and 40 cycles of 95 °C for 15 s and 60 °C for 1 min using Applied BioSystems ViiA 7 System. The Human MALAT1 mRNA relative expression (RQ) to GAPDH was calculated. Each sample was run in duplicate.

### HDF scratch assays

Human Dermal Fibroblasts-adult (HDF-a) cells were purchased from ScienCell, Carlsbad, CA, # 2320). HDF-a were from adult human skin and were negative for HIV-1, HBV, HCV, mycoplasma, bacteria, yeast and fungi. Cells were subcultured in Fibroblast Medium (ScienCell, # 2301) until approximately 70% confluent and media changed every other day until the cells were about 85% confluent.

For performing a wound healing scratch assay, we used the Ibidi Culture-insert 2 well (Ibidi USA, Fitchburg, WI, # 80,209). The sterile 2-well insert was placed into each well of a 12-well plate; HDF-a cell suspension of about 3.6 × 10^5^/ml, was added (70 µl of cell suspension) to each well of the plate with inserts. Cells were cultured at 37 °C with 5% CO_2_ as usual. After 24 h, inserts were removed, and the plate was rinsed with PBS to remove cell debris. Then cells were preincubated with mitomycin C (10 µg/ml) for 2 h to block further proliferation so that only migration was followed.

Isolated exosomes 20 µg/ml (protein) were then added to wells. Basic fibroblast growth factor (bFGF, Gibco, # 13,256–029) was used as the positive control, 20 ng/ml. Wound healing assay photo images were acquired at 0, 8, 26, and 48 h. Images were analyzed by Ibidi Metavi Wound Healing Analysis Automatic Cellular Analysis System and by counting cells that had migrated into the scratched field. Data from each time point and treatment were analyzed by one-way ANOVA with *p* < 0.05.

### Mitochondrial stress test

Human Dermal Fibroblasts-adult (HDF-a) cells were cultured in a T-75 flask following the manufacturer’s instructions. For the Mitochondrial Stress test the HDF-a, 5.5–6 × 10^3^ cells, were plated into wells of a poly-L-lysine coated XFp cell culture miniplate with 80 µl of growth medium. In two of the 8 wells, no cells were plated for background correction. Sterile water (400 µl) was added to each chamber of the moat. Cells grew overnight in a cell culture incubator. Cells were then treated with exosomes prior to the stress test. Exosomes (20 µg protein/ml) were added to the wells with serum free medium, and cultured in a CO_2_ incubator for 2–24 h. The Seahorse Mito Stress Tests used the Agilent Seahorse XFp Extracellular Flux Analyzer. Oxygen consumption rate (OCR) was measured using the mitochondrial stress test kit (Agilent Technologies, Santa Clara,CA, # 103,010–100) following instructions. Agilent Seahorse XF DMEM media (# 103,575–100) containing 10 mM glucose, 2 mM L-glutamine, and 1 mM sodium pyruvate were used prior to the assay and the compounds for loading the sensor cartridge ports for the Agilent Seahorse XFp analyzer were: 1.0 μM of Oligomycin (Port A), 1 μM of FCCP,carbonylcyanide p-(trifluromethoxy)phenyl-hydrazone, (Port B) and 0.5 μM of Rotenone/Antimycin A (Port C).

The Agilent Seahorse XF cell Mito Stress Test report generator automatically the Agilent Seahorse XFp Cell Mito Stress Test parameters from WAVE™ data that was exported to Excel. Exosome preparations were tested in 3–4 separate assays. Significance was tested using a two-tailed Student’s t test (Microsoft Excel) with at least three independent biological replicates (n), with the Mann–Whitney U test or with one-way ANOVA as indicated (GraphPad Prism) (*p < 0.05, **p < 0.01, and ***p < 0.001).

### sEV size and charge

sEV size was determined using Malvern Nano Series Zetasizer Nano ZS90. A sEV aliquot of 50 µl was added to 450 µl nuclease-free water and mixed in a cuvette for determining the size of sEV in the instrument.

## Results

### Ceramide responsive cis-elements in MALAT1

A putative CRCE in the MALAT1 promoter is predicted by bioinformatics. The longest MALAT1 transcript from human is 8708 nt in length [GenBank: NR_002819.2], and the transcriptional start site was defined [18]. Inspection of the MALAT1 sequence reveals several CRCEs core motifs as shown in Table 1. A core motif is defined as the sequence within the RNA recognition motif that are found in RNA binding proteins controlling the production, maturation, localization, translation, and degradation of cellular RNAs. Here, we did not identify the RNA binding domain protein (RBD), but inspected the MALAT1 sequence for motifs resembling those described previously for a ceramide responsive RBD protein[11]. Although we did not identify a full-length motif, we were able to identify seven motifs with six to nine base matches. Most RBDs make contact with 3–5 contiguous RNA bases [19]. These motifs were not found in GAS5 or in HOTAIR, other lncRNAs in exosomes[6]**.**

**Table 1:** CRCE elements

A. Ceramide-responsive RNA cis-elements (CRCE) identified by (11):
gagggaggcaggcga
ggaaaaagaggaacg
B. CRCE core motifs present in MALAT1 (*homo sapiens*):
101- aaggcaggt
2761- aggaaaagag
4841- tgatgagggagg
6861- tggtgggagg
7701- aggggaggga
8491- ttggggaggt gggaggtaac

CRCE were identifie by manually searching the transcripot of MALAT1 in MSWord

### Inhibition of ceramide synthesis lowers MALAT1 levels in ADSC and sEVs

**Fig. 1:**
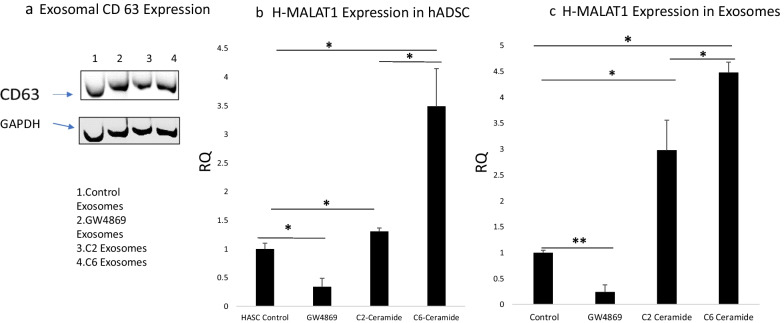
MALAT1 levels in hADSC and exosomes after treatment with modulators of ceramide synthesis. **a** Expression of CD63 by exosomes from hADSC treated with modulators of ceramide synthesis. **b** MALAT1 in hADSC RNA in control cells, and cells treated with GW4869, C2- and C6- ceramide. *p < 0.05 as determined by Graph Pad one-way ANOVA. **c** Exosomes were isolated and MALAT1 expression was measured by RT-PCR in CM from treated cells. *p < 0.05 and **p < 0.01 as determined by Graph Pad one-Way ANOVA followed by Friedman’s test

We questioned how MALAT1 was recruited and packaged into exosomal vesicles via CRCE. Sphingomyelin (SM) is hydrolyzed into phosphorylcholine and ceramide (Cer) by a family of sphingomyelinases that function at acidic, neutral, or alkaline pH [15]. Sphingomyelinases are specifically associated with cellular compartments [16]. Previous studies showed that SM hydrolysis and Cer formation participate in endosomal sorting complex required for transport (EXCRT)-independent biogenesis of intraluminal vesicles inside MVBs, the vesicles that are released as exosomes upon MVB fusion with the plasma membrane [2]. A lipid based mechanism for exosome formation was proposed. Hence, we examined release of exosomes here by treating stem cells with GW4869, an inhibitor of n-SMase 1 and n-SMase 2 which reduces long chain ceramides. Exosomal packaging of MALAT1 was demonstrated previously using GW4896 [17]. Here, human ADSC were treated with GW4869 while CM was collected to provide hADSC, exosome and microvesicle MALAT1 analysis by qPCR. All preparations of exosomes and microvesicles exhibited the same level of CD63, an exosomal surface marker (Fig. 1A). Figure 1B demonstrates that MALAT1 concentrations in the hADSCs were reduced 75% by GW4869 compared to control, untreated hADSC. sEV MALAT1 content was also significantly diminished by treating the hADSC with the nSMNase inhibitor, GW4896 (Fig. 1C).

Our finding is novel in that ceramide synthesis appears to be involved in both the generation and packaging of MALAT1 into exosomes.

### C2- and C6-ceramide treatment of hADSC increase MALAT1 levels

To further define ceramide function in MALAT1 packaging, we treated ADSC with C2- and C6-ceramides during collection of CM. These precursors can incorporate into long chain ceramides by several synthase pathways. C2-ceramide, stimulates the de novo pathway of synthesis, increased the cellular amount of MALAT1 by 30% compared to control cells (Fig. 1b), but increased exosomal MALAT1 by threefold compared to control exosomes (Fig. 1c). These sEVs however, were larger in size than exosomes and considered to be microvesicles, corresponding in size to those from GW4869 treated cells (Table 2). For comparison, C6-ceramide was also used since it is known to incorporate into different synthase pathways than C2-ceramide. C6-ceramide increased cellular MALAT1 3.5-fold and exosomal MALAT1 content by 4.5-fold. The size of the sEV secreted corresponded to exosomes (Table 2). All sEV preparations had negative charges as expected (data not shown). The C6 effects indicate that potentially two synthase pathways are involved in MALAT1 transcription, cellular retention and packaging into exosomes. Hence, ceramide synthesis increased packaging of MALAT1 in proportion to its intracellular levels.

**Table 2:** sEV size

Cell treatment	Size(d.nm)	St Dev (d.nm)
Control	149.2	30.35
GW4869 inhibitor	301.9	80.99
C2-ceramide	308.9	61.56
C6-ceramide	136.7	30.29

sEVsize variation based on treatment of hADSC with ceramide synthesis modulators. sEVof < 150 nM are considered exosomes

### Human dermal fibroblast migration responded to ceramide and MALAT1 containing exosomes in scratch assays

**Fig. 2:**
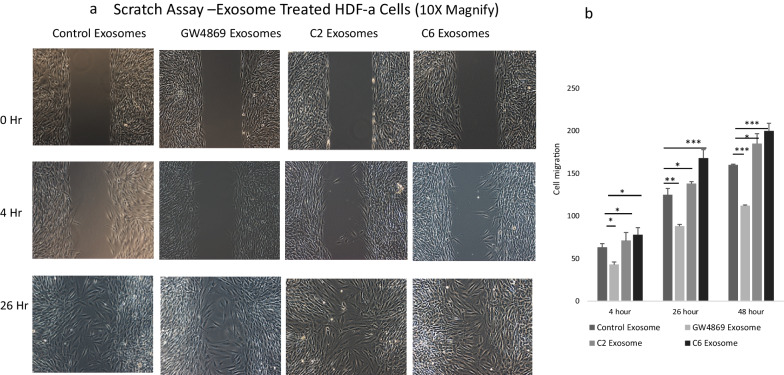
Cell migration assays using HDF-a treated with exosomes as indicated. **a** The cell free gap was created by Ibidi inserts, and images of cell migration were captured at times indicated. **b** Migrating cells were counted by Olympus CellSens software. Data are expressed as mean + SEM and data was analyzed using Graph Pad one way ANOVA with p-tests the Friedman test. *p < 0.05 and **p < 0.01 were considered to be statistically significant compared to control exosomes

Given that ceramide synthesis modulated packaging of MALAT1, we examined sEV function on cellular migration by treating HDF with the different sEV isolates using a modified scratch assay where myriocin C pre-treatment blocked cell proliferation so that only migration was observed. Control exosomes increased cell migration (Fig. 2a). sEV from GW4869-treated cells, containing significantly lower levels of MALAT1, slowed cell migration compared to control exosomes. However, migration resulting from sEV collected from C2-treated stem cells stimulated migration even more than the control exosomes but less than C6-treated cell derived exosomes with higher levels of MALAT1 (Fig. 2a,b). This would indicate that the larger sEV could also be therapeutic if they contained MALAT1. The increased levels of MALAT1 in sEV (4- to 5- fold over control exosomes) did not increase cell migration in a dose responsive manner, however, but migration was significantly faster as shown in Fig. 2b. This suggests that there is an optimal level of MALAT1 utilized to increase cell migration.

### Exosome treatment of HDF improves mitochondrial function

**Fig. 3:**
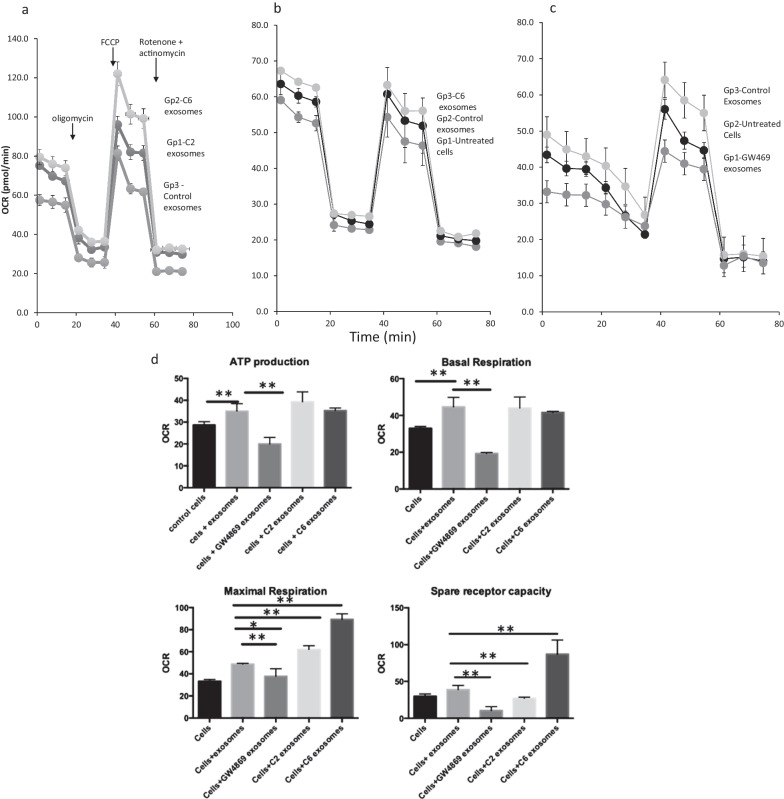
Seahorse Mitocondrial Stress Test performed on HDF-a treated with exosome isolates. **a–c** Mitochondrial respiration is indicated by OCR was detected under the conditions of oligomycin (1 µM, the uncoupler, FCCP (1 µM) and the electron transport inhibitor rotenone (0.5 µM) injection as shown by arrow in 3A. Groups (gps) are identified in their order within the graphs of the grey shaded plots. **d** ATP production, Maximal respiration, Basal respiration, and spare receptor capacity are compared between the control cells and cells treated with exosome isolates. *p < 0.05; and **p < 0.01 as determined by Student t-test

One aspect of exosomes and microvesicles that has not been investigated is their potential for increasing mitochondrial function. Increased cellular ATP is required for wound healing, and the fine tuning of reactive oxygen species (ROS) is integral to this process. We studied the effect of sEV from GW4869-treated hADSC (GW4869- sEV) on HDF subjected to the Mito Stress Test. GW4869-sEV, with very little MALAT1, resulted in decreased OCR (pmol/min/ 5,500 cells) detected as ATP production by 40% and also decreased basal and maximal respiration (Fig. 3c,d).

In another Mito Stress Test, control, untreated exosomes increased HDF basal OCR by 45% compared to untreated HDF (Fig. 3c). The overall maximal respiration from control and C2- and C6- sEV was increased 50% with a significant difference between control and Cer-treated exosomes for ATP production. C2-and C6-ceramide sEV contained more MALAT1 as shown in Fig. 1c. The increased MALAT1 in C2- and C6- exosomes did not significantly alter ATP production or basal respiration more than the control exosomes suggesting that MALAT1 was important for increasing parameters related to mitochondrial reactive oxygen species (ROS).

Here, the first measurement is the basal OCR measured in cells prior to injection of oligomycin (Fig. 3a). Oligomycin treatment, the ATP synthase inhibitor, revealed that control exosomes increased ATP synthesis and GW4869-sEV reduced ATP. Higher MALAT1 levels in C2- and C6-sEV also increased ATP production (Fig. 3a, b, d), but not significantly from control exosomes. Maximal respiration, calculated after FCCP-treatment, a potent uncoupler of oxidative phosphorylation, and Antimycin A + Rotenone, inhibitors of electron transport chain, was increased by control exosomes, C2- and C6- sEV (Fig. 3b, d). This suggests that maximal OCR, estimated by FCCP compared to basal respiration, demonstrated mitochondria using less than the maximal rate of electron transport that can be supported by substrate from the cells. The difference between basal and maximal respiration is the spare or reserve capacity of energy. Whether the HDF utilize the maximal electron transport activity for ATP synthesis depends on the capacity of the oxidative phosphorylation system which may limit a response. In the spare receptor capacity, we see the difference between C2- and C6- sEV and project that stimulation of the two ceramide synthase pathways alter/increase other components of the sEV (Fig. 3d).

## Discussion

The packaging of lncRNAs such as MALAT1 with at least 7 partial or core motif CRCE was regulated by the C2- and C6- synthase pathways when stem cells were exposed to exogenous short chain ceramides. C2-treatment altered the size of the sEV, but C6-treatment did not. These sEV, or exosomes, increased the migration rate of HDF in scratch assays, and also increased HDF maximal respiration by 40–50% in mitochondrial stress assays. Increased mitochondrial function is necessary for the healing of dermal wounds as represented by the scratch assay.

Overall, the packaging of MALAT1 into ADSC exosomes and microvesicles appears to be regulated by three ceramide synthase pathways in different manners. A loss of function in a sub-population of exosomes treated with siRNA for MALAT1 decreased the ability of cells to migrate in our initial studies [9]. However, MALAT1 levels increased up to 4.5-fold in C6-treated exosomes as determined by comparison to control exosomes, but did not further improve HDF migration, basal respiration and ATP production. This upper limit of MALAT1 function may be due to a protective mechanism from too much of a lncRNA that is often associated with cancer [20]. It may also be due to a saturating level intracellularly.

The finding that overexpression of MALAT1 via ceramide synthase pathways is important for wound healing is novel. Sphingolipids are known to exert critical roles in disease states. Mitochondrial ceramide generation and transport of ceramide to mitochondria regulate its function as shown by the increase in exosome function from ceramide and MALAT1 enriched exosomes[21].

In our study, exogenous C2- and C6-derived ceramides altered maximal mitochondrial respiration of HDF exposed to sEV. The effect of C2-derived ceramide was shown to alter gene expression by inhibition of ROS production in invasive cells via the inhibition of matrix metalloproteinase gene expression [21]. However, maximal respiration is also associated with modulation of ROS and this aids in wound healing via fibroblast mitochondrial affecting genes central to wound healing [22]. Our results show that C6-derived exosomes promoted a significant increase in cell migration at later time points to support this. C2-derived sEV application also demonstrated a higher rate of scratch closure, but not significantly different from control exosomes which may indicate specificity for exosomes in wound healing vs sEV.

The fact that C2- and C6-derived ceramides both increased MALAT1 levels is not surprising, as MALAT1 contains a CRCE in its promoter determined by bioinformatics. Ceramide is generated by three metabolic pathways: de novo synthesis, sphingomyelin hydrolysis, and the salvage pathway [23]. The fact that the inhibitor of sphingomyelin hydrolysis, GW4869 blocked MALAT1 transcription, packaging and secretion suggests a pivotal role for ceramide synthesis in packaging of sEV lncRNA cargo. C2- and C6-ceramide treatment of cells is known to increase long chain (14–26 carbons) ceramide concentrations via a set of six ceramide synthases with some having overlapping specificity and others synthesizing C18 ceramide, C18-22 ceramide and C22-24 ceramide while one yields C26-ceramide and above. The salvage pathway produces ceramide from catabolism of complex sphingolipids yielding sphingosine[23].

Ceramide synthases are cell/tissue specific, and usually found in the endoplasmic reticulum, but also in mitochondria where they are components of inner and outer mitochondrial membranes. C2-ceramide stimulates mitochondrial complex IV activity [24]. Other ceramides inhibit complex III [25]. The effects of different ceramides has varying effects on mitochondrial membrane potential. C6-ceramide caused mitochondrial depolarization [26]. Hence, the effects of the C2-ceramide precursors could have effects on sEV size that is transferred to the HDF in addition to MALAT1 which may carry ceramides into sEV and subsequently transfer it to HDF mitochondria.

MALAT1 was found in the mitochondria of HepG2 cells [27, 28]. However, in these studies MALAT1 originated in the nucleus and was found enriched in mitochondria where it interacted with mitochondrial proteins. Our study suggests that exosomal MALAT1 was utilized by HDF mitochondria to alter their function.

The downregulation of exosomal MALAT1 levels by GW4896-treatment of stem cells shown here resulted in the inhibition of mitochondrial ATP production, basal and maximal respiration. Whether the effect was due to MALAT1 depletion, the size of the sEV, or other factors altered by inhibition of ceramide synthesis is unknown. However, the role of MALAT1 in mitochondrial function was established previously, and our results demonstrate a role for the lncRNA in wound healing and mitochondrial function of noncancerous cells. Studies of MALAT1 function often focus on the overexpression of the lncRNA in cancer cells. Here, we are focusing on the function of this abundant non-coding RNA when packaged into exosomes derived from healthy stem cells. The function of putative CRCE in lncRNA has not been reported. There are certain limitations to our study in that CRCE motifs remain putative since site-directed mutagenesis of the long MALAT1 transcript was not possible. Also, the levels of ceramide species were not measured.

The original element contained 15 nucleotides. Here, we have found putative core elements of 6–8 nucleotides. The occurrence of seven of these elements in MALAT1 while other exosomal lncRNA such as HOTAIR and GAS5 contain only one partial sequence each, suggests that these elements are unique to certain lncRNAs such as MALAT1 and this facilitates its packaging into exosomes. Little is known about how lncRNA are selected as cargo for exosomes. This finding has therapeutic implications for ceramide synthesis by stem cells.

## Conclusions

Stem cells are a rich source of exosomes. The cellular packaging of MALAT1 into exosomes was regulated by ceramide synthase pathways. Overexpression of MALAT1 via ceramide synthase was important for cell migration in scratch assays. Since the inhibitor of sphingomyelin hydrolysis, GW4869, blocked MALAT1 transcription, packaging, secretion and cell migration, this suggests a pivotal role for ceramides in inclusion of lncRNA cargo in exosomes. GW4869 downregulation of exosomal MALAT1 content also resulted in the inhibition of mitochondrial ATP production, basal and maximal respiration. The fact that MALAT1 contains CRCE may explain how ceramide regulates its transcription and packaging via ceramide dependent activities.

## Data Availability

All data and materials are available upon request.

## Abbreviations

ADSC: Human adipose derived stem cells
Cer: Ceramide
CM: Conditioned media
CRCE: Ceramide response cis-elements
sEV: Small extracellular vesicle
HDF: Human dermal fibroblasts
lncRNA: Long non-coding RNA
MALAT1: Metastasis-associated lung adenocarcinoma transcript 1
n-SMase: Neutral sphingomyelinase
OCR: Oxygen consumption rate
RBD: RNA binding domain
ROS: Reactive oxygen species
